# Supplementary material for: Panpipes: a pipeline for multiomic single-cell and spatial transcriptomic data analysis
Source: Genome Biol. 2024 Jul 8;25:181. doi: 10.1186/s13059-024-03322-7 (PMC11229213; doi:10.1186/s13059-024-03322-7)
Supplement: Supplementary file 1 — Additional file 1: Figures S1 and S2. [file 13059_2024_3322_MOESM1_ESM.pdf]

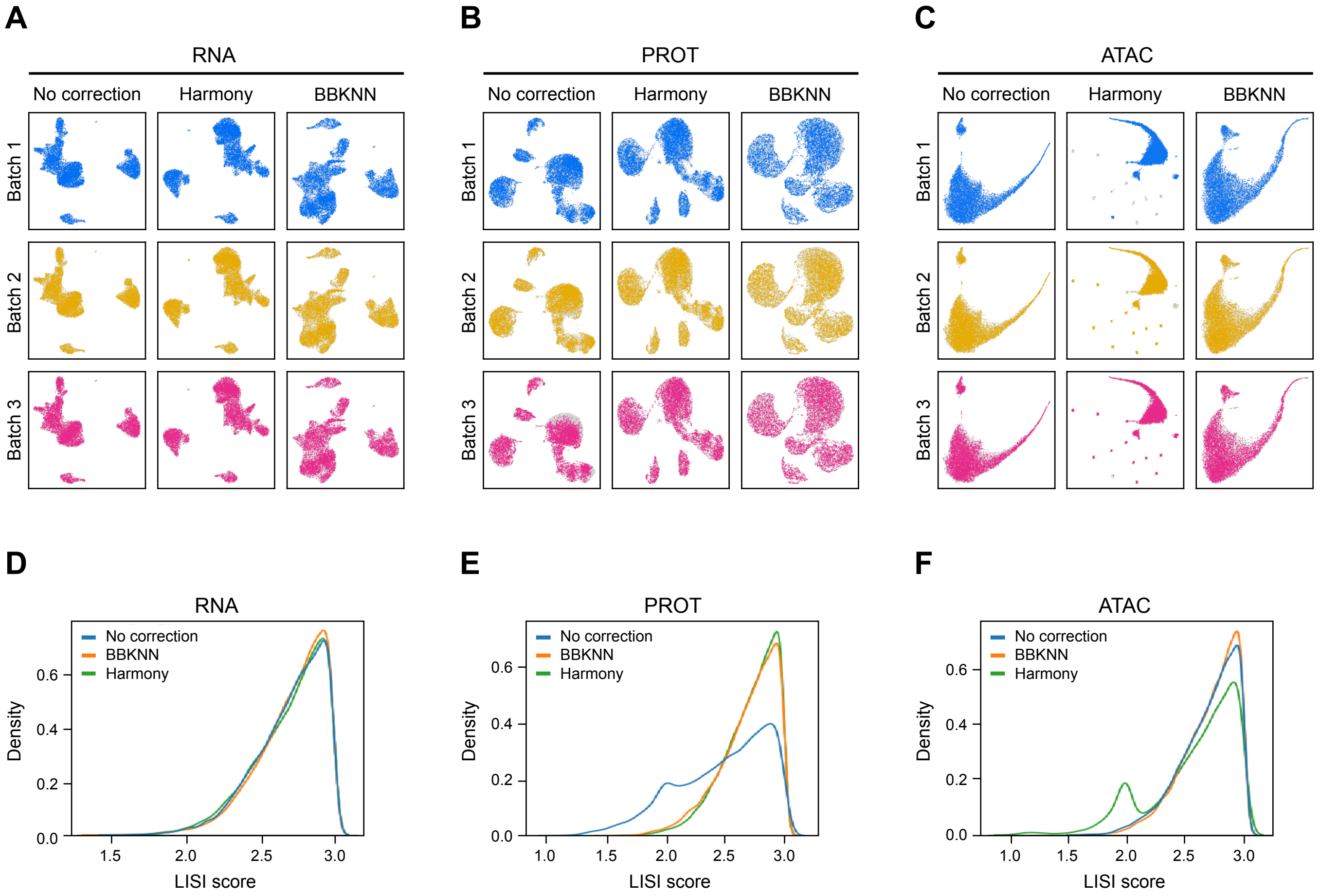

Figure S1. Panpipes integration workflow enables evaluation of batch correction of different individual modalities. UMAPs showing individual batches (batch 1, blue; batch 2, ochre; batch 3, pink) with no correction or after unimodal Harmony or BBKNN batch correction for individual **(A)** RNA, **(B)** ADT cell-surface protein (PROT), and **(C)** ATAC modalities from the trimodal TEA-seq dataset. LISI score distribution for no batch correction (blue) or unimodal Harmony (green) or BBKNN (orange) batch correction for individual **(D)** RNA, **(E)** PROT, and **(F)** ATAC modalities from the trimodal TEA-seq dataset.

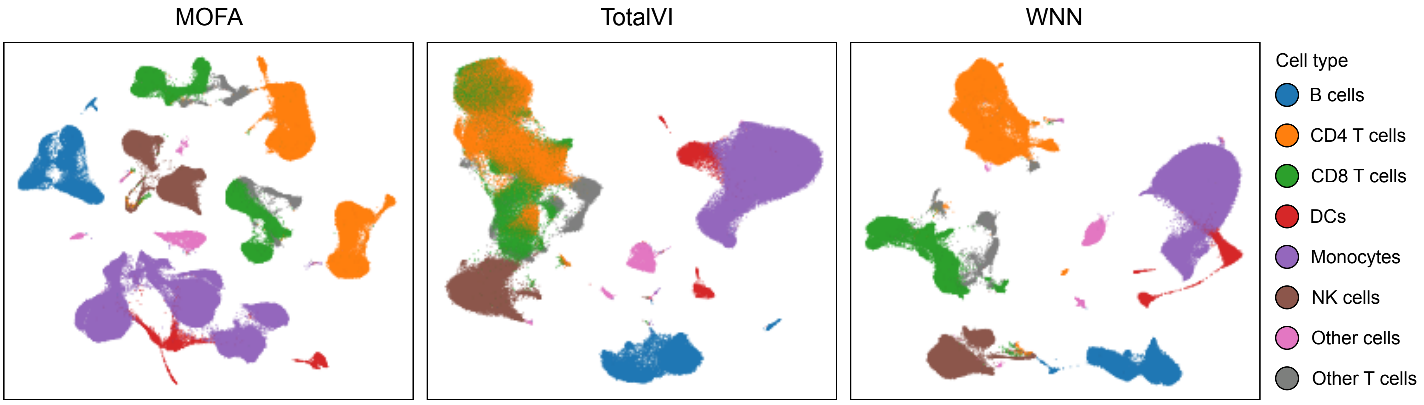

Figure S2. Panpipes integration workflow enables visualisation and evaluation of integration and batch correction in a biological context.
UMAPs showing the integration of a previously annotated, available PBMC CITE-seq dataset after utilisation of MOFA, totalVI, and WNN. The distribution of the PBMC subsets in the UMAP plots can be visually inspected, taking into consideration the cell type labels. DCs: dendritic cells; NK: natural killer.
