# Supplementary material for: Panpipes: a pipeline for multiomic single-cell and spatial transcriptomic data analysis
Source: Genome Biol. 2024 Jul 8;25:181. doi: 10.1186/s13059-024-03322-7 (PMC11229213; doi:10.1186/s13059-024-03322-7)
Supplement: Supplementary file 2 — Additional file 2: Review history. [file 13059_2024_3322_MOESM2_ESM.docx]

Review History

**First round of review**

**Reviewer 1**

**Were you able to assess all statistics in the manuscript, including the appropriateness of statistical tests used?**

There are no statistics in the manuscript.

**Have you been able to install and test the software?**

No

**Comments to author:**

The authors introduce "Panpipes," a software for multiomic single-cell data analysis. Panpipes automates the analysis of multimodal single-cell datasets by implementing established open-source software and state-of-the-art methods. It performs quality control, preprocessing, integration, clustering, and reference mapping. The software is flexible, allowing unimodal and multimodal integration with or without batch correction and offers reference mapping for annotating user cell types from reference datasets. The reference mapping functionality thereby provides a useful tool to seamlessly annotate user's cell types from reference datasets.
However, we perceive Panpipes as an incremental advance in a field that already has dedicated workflows for data integration and evaluation. Existing tools like Seurat also integrate multimodal single-cell datasets, raising questions about why the authors did not benchmark Panpipes against these pipelines.
Considering the incremental nature of the study, it may be more appropriate to present it as a review or protocol paper on best practices for multiomics analyses using existing tools. However, even under that scenario, certain changes are necessary to enhance software accessibility and benchmarking.

The following comments are therefore made with this in mind.

Major
1) The main advance of this study is the establishment of the integrated pipeline. However, given that its deployment and installation is still rather complex, it seems not suitable for an inexperienced user for which it seems to be catered. For example, customizing the pipeline requires changing non-trivial yaml and other config files. We therefore believe that this tool would be best suited for experienced bioinformaticians or sequencing/bioinformatics services platforms, but not for lay users.
2) We tried running it on our own local system, and -after fixing some dependencies and other bugs- it failed at first step of "panpipes qc_mm make full -- local" with 2 errors:
a. "/etc/bashrc: line 12: BASHR CSOURCED: unbound variable"
b. "TypeError: argument of type 'NoneType' is not iterable"
So, we could not test the pipeline as we could not trace the source of these errors. In addition, we do not understand why the tool tries to access the /etc/bashrc file?
Some possible suggestions may include:
a. Offer the pipeline in an image such as docker or singularity which will ensure the reproducibility and ease of access for all the users.
b. Reformat or wrap the pipeline as a Python and/or R package.
2) The authors presume that the pipeline's scalability, resources and time requirements allow the user to deal with big datasets in HPC and locally. Nevertheless, they do not offer benchmarking where time, resources and scalability could be evaluated. We therefore suggest:
a) Comparison to already existing workflows such as Seurat
b) Resource and time scalability for differently sized datasets (number of cells)
3) The study lacks sufficient biological context and guidance for users. More information and examples are needed, particularly in the Integration step. Guidance should explain the benefits of different algorithms and provide comparisons with already published methods.
4) The Integration step constitutes a nice repertoire of different algorithms for processing differential modality datasets. Nevertheless, it lacks guidance and explanation or assessment for establishing a decision to understand or determine which one is providing an advantage.
a) Guide and explain the user which algorithm to use, providing some inputs in the "Integration" step, with comparisons against other datasets already used for this, such as https://doi.org/10.1016/j.cell.2021.04.048.
b) Comparing against already published methods and show the benefits of the selected algorithms.

Minor
* Figure 1 may confuse readers as it shows convergence of all modalities in the Integrated MuData with kNN graph, while the pipeline allows for unique integration methods for each modality combination.
* The responsibility for pipeline maintenance and updates is not discussed.
* Arguments like GEX or RNA in the yaml file could create confusion and require further description.

**Reviewer 2**

**Were you able to assess all statistics in the manuscript, including the appropriateness of statistical tests used?**

There are no statistics in the manuscript.

**Were you able to directly test the methods?**

Yes

**Comments to author:**

Panpipes is a wonderful and timely tool that provide common workflows for single-cell multi-omics data analysis. The workflows are carefully selected, with the overall framework comprehensively designed. Overall, this collection of workflows will be welcomed by bioinformaticians.
The review wonders whether providing more information on the following will help highlight the strength and usefulness of Panpipes and facilitate the use of the tool:
* How good the Panpipes tool follows the FAIR principles? It will be wonderful to summarize key functionalities according to the four FAIR principles.
* Data standards. A summary about the current data standards and metadata standards widely used in biomedical consortia of single cell omics data and by funding agencies and how Panpipes supports such standards will be helpful. Especially, starting from this year, NIH requires all proposals and funded projects should follow the new Data Management and Sharing policy. Therefore, many researchers start to examine whether tools are compatible with such requirements. Providing explicit descriptions will help researchers to decide whether they want to use Panpipes.
* Flexibilities of switching specific functionalities to different tools. Panpipes claims that incorporating new omics types is supported, due to the modularity of the system. Meanwhile, since there are so many new tools emerging, just wonder whether authors can describe what interface and design allow Panpipes to adapt new tools, and to specify which tools can be easily customized to replace the current Panpipes tools by the end users, and which needs significant amount of coding. This is particularly important since some choices of tools by Panpipes are different from what my lab and my peers use. I fully respect Panpipes' choices of tools. Meanwhile, whether bioinformaticians can easily use Panpipes to cover tools that we regularly use is a major determinant of whether my lab will use Panpipes.
* The support to spatial omics data. Many single-cell omics tools have recently released their spatial omics pipelines. Here are some examples: 1) Seurat pipeline was not initially developed for spot-level ST data, but for single-cell RNA-Seq data. They have officially claimed that their pipeline can be used for both spot-level ST data (https://satijalab.org/seurat/articles/spatial_vignette.html) and the single-cell ST data (https://satijalab.org/seurat/articles/spatial_vignette_2.html). Scanpy also released the official example for single cell ST data (MERFISH data, https://scanpy-tutorials.readthedocs.io/en/latest/spatial/basic-analysis.html#MERFISH-example). Just wonder whether Panpipes considers including such supports, or would like to leave it to future versions? Extending supports to spatial omics data can be achieved in a short time period (i.e., during revision time). It will significantly increase the impact of this useful tool.
* Panpipes provides amazing visualization functions and other functions. However, this capacity has not been fully demonstrated in the github documentations. The reviewer strongly recommends including a series of vignettes in github to demonstrate the visualization functionalities as well as how to use Panpipes in other common use cases. The reviewer, and maybe other peers, are so "spoiled" by the decent documentations provided with other bioinformatics tools, and thus expect to have ready-to-use vignettes to start with when using Panpipes in our research. For example, the scanpy tutorials (https://scanpy.readthedocs.io/en/stable/tutorials.html) are so helpful for my students and colleagues to follow. Could Panpipes consider providing tutorials and vignettes at this level and degree please?

**Authors Response**

**Point-by-point responses to the reviewers’ comments:**

**GBIO-D-23-00361** **“Panpipes: a pipeline for multiomic single-cell and spatial transcriptomic data analysis”**

**Reviewer Rebuttal**

**Reviewer #1:** *The authors introduce "Panpipes," a software for multiomic single-cell data analysis. Panpipes automates the analysis of multimodal single-cell datasets by implementing established open-source software and state-of-the-art methods. It performs quality control, preprocessing, integration, clustering, and reference mapping. The software is flexible, allowing unimodal and multimodal integration with or without batch correction and offers reference mapping for annotating user cell types from reference datasets. The reference mapping functionality thereby provides a useful tool to seamlessly annotate user's cell types from reference datasets.*

*However, we perceive Panpipes as an incremental advance in a field that already has dedicated workflows for data integration and evaluation. Existing tools like Seurat also integrate multimodal single-cell datasets, raising questions about why the authors did not benchmark Panpipes against these pipelines. Considering the incremental nature of the study, it may be more appropriate to present it as a review or protocol paper on best practices for multiomics analyses using existing tools. However, even under that scenario, certain changes are necessary to enhance software accessibility and benchmarking.*

Based on the reviewer’s comments we have recognised that we had not originally clarified the difference between a pipeline (Panpipes) and a framework (e.g. Seurat), which may have been a source of confusion. Hence, we have amended the manuscript to better explain this difference and what Panpipes offers further to frameworks, and we briefly detail the key differences below. We have also expanded the scope of the pipeline by adding spatial transcriptomic analysis workflows, and we provide new documentation including tutorials to facilitate users with installation and pipeline running, available at <https://panpipes-pipelines.readthedocs.io/en/latest/>.

Seurat is an analysis framework, whilst Panpipes is a pipeline built with a Workflow Management Language orchestrating analyses with Scanpy, muon and squidpy (which are themselves analysis frameworks). Several manuscripts have already focused on benchmarking of frameworks such as Seurat, scanpy and muon (e.g. Wolf *et al*. 2018 *Genome Biology*; Bredikhin *et al.* 2022 *Genome Biology*; Pullin and McCarthy 2022 *bioRxiv*), which is hence outside of the scope of this manuscript. Briefly, Seurat leverages R’s statistical capabilities, whilst Scanpy uses Python’s machine learning libraries; each framework has its own inherent strengths and they cater to different programming communities (largely R versus Python users). They also use distinct data structures (SeuratObject, AnnData and MuData) that are not directly interchangeable, which can complicate direct cross-framework comparisons. By extension, comparing an analysis framework like Seurat to a pipeline like Panpipes which is built with components from frameworks distinct to Seurat can lead to a misinterpretation of their relative roles and functionalities. Nevertheless, where some direct comparisons were possible between Seurat and Panpipes we have run these (as detailed in comment 4 below).

As a pipeline, Panpipes has several key benefits which we believe will appeal to single-cell analysts (and particularly those who are Python users). Other single-cell pipelines (rather than frameworks) are scFlow (Khozoie *et al.* 2021 *bioRxiv*) and scrnaseq (<https://github.com/nf-core/scrnaseq/blob/2.3.2/README.md>), however these only ingest unimodal single-cell RNAseq data, whilst Panpipes is designed for uni- or multi-modal data analysis and now also includes workflows for spatial transcriptomic analysis. It provides reproducibility afforded by the encapsulation of all the steps of data processing and analysis (which also enables FAIR principle compliance). It has the capacity for parallelisation (which is critical when running and testing different algorithms and which facilitates scalability), and for the optimisation of resource utilisation via distributed computing environments. Moreover, Panpipes has the capacity for customisation and ease of maintenance, given its modularity. To further facilitate this, we deliberately chose to build Panpipes on scanpy and muon, so as to include it in the scverse ecosystem.

As scverse is interoperable with Bioconductor (R ecosystem), our choice of using scverse will ensure that Panpipes is future-proof, enabling full interoperability thanks to the conversion components implemented as part of the scverse community efforts.

*The following comments are therefore made with this in mind.*

**Major**
1)      *The main advance of this study is the establishment of the integrated pipeline. However, given that its deployment and installation is still rather complex, it seems not suitable for an inexperienced user for which it seems to be catered. For example, customizing the pipeline requires changing non-trivial yaml and other config files. We therefore believe that this tool would be best suited for experienced bioinformaticians or sequencing/bioinformatics services platforms, but not for lay users.*

We have clarified the text in the main manuscript accordingly. Our intention is not for Panpipes to provide a user-friendly interface for lay users with no bioinformatics skills; rather we envisage that outside of experienced single-cell analysts or services platforms, Panpipes could be of utility for those bioinformaticians who are relatively new to the single-cell field and who would thus benefit from a set of streamlined workflows that provide the flexibility to test and compare different algorithms for multimodal dataset analysis. In addition, we have now added documentation and tutorials to help facilitate even experienced users in implementing and running Panpipes as detailed in <https://panpipes-pipelines.readthedocs.io/en/latest/>.

2)      *We tried running it on our own local system, and -after fixing some dependencies and other bugs- it failed at first step of "panpipes qc_mm make full -- local" with 2 errors:
a.      "/etc/bashrc: line 12: BASHR CSOURCED: unbound variable"
b.      "TypeError: argument of type 'NoneType' is not iterable"*

*So, we could not test the pipeline as we could not trace the source of these errors. In addition, we do not understand why the tool tries to access the /etc/bashrc file.*

We apologise for this problem and thank the reviewer for spotting these issues which were due to a blank field in the pipeline.yml for the qc_mm workflow which we have corrected. The tool trying to access the /etc/bashrc file is a feature of the workflow language we are employing but will not hinder pipeline usage. In recognition of the need to facilitate users with pipeline installation, as noted above we now provide relevant step-by-step tutorials.

3)	*Some possible suggestions may include:
a.      Offer the pipeline in an image such as docker or singularity which will ensure the reproducibility and ease of access for all the users.
b.      Reformat or wrap the pipeline as a Python and/or R package.*

We thank the reviewer for suggesting these options to fix the issues they encountered. We have decided to follow the second option such that further to the documentation now supplied for installation, Panpipes is now installable via PyPi with `pip install panpipes` as a Python package, and we provide instructions to install the required packages via `panpipes install_r_dependencies`.

4)      *The authors presume that the pipeline's scalability, resources and time requirements allow the user to deal with big datasets in HPC and locally. Nevertheless, they do not offer benchmarking where time, resources and scalability could be evaluated. We therefore suggest:
a.      Comparison to already existing workflows such as Seurat
b.     Resource and time scalability for differently sized datasets (number of cells)*

As we have noted above, Panpipes is a pipeline (built with components from different frameworks and which allows parallelisation of analyses), whilst Seurat is an analysis framework (where different analyses of the same dataset need to be run serially), such that direct comparisons can lead to misinterpretation of the role and functionality of Panpipes as compared with Seurat. Nevertheless, we have run a benchmarking analysis using the dataset indicated by reviewer (and also referenced in comment 5), subsampling down to different number of cells and with different resources according to the size of the dataset. We have used the Seurat v5 package (which was released after our submission) and followed the tutorial provided at https://satijalab.org/seurat/articles/seurat5_integration. This comparison demonstrates the ability of Panpipes to run multiple methods in parallel with runtime increasing with cell numbers, as expected.

In all comparisons, Panpipes’s performance is better than Seurat in terms of runtime and optimal usage of compute resources thanks to parallel computation (see plot below). We wish to note that for the Seurat benchmarks run on GPUs, all the steps are run on GPUs. For Panpipes the current implementation preferentially spawns most of the computation on CPUs rather than GPUs (in this benchmark only scVI is run on GPU). The reason for this is that the current implementation reflects the structure of current HPCs, which normally have a limited number of GPUs available. In a future release we will invert this behaviour, running all computations on GPUs whenever possible. We anticipate that this would reduce Panpipes run times even more, such that these would likely be substantially faster than when running Seurat on GPUs.

In addition, we have performed a comparison of Panpipes run times and resource (CPU/GPU) utilisation for differently sized datasets, and this information is summarised in the new Results Benchmarking section and corresponding Figure 5. The code used for the benchmarks is available at <https://github.com/DendrouLab/panpipes-benchmarks>.

5)      *The study lacks sufficient biological context and guidance for users. More information and examples are needed, particularly in the Integration step. Guidance should explain the benefits of different algorithms and provide comparisons with already published methods.
The Integration step constitutes a nice repertoire of different algorithms for processing differential modality datasets. Nevertheless, it lacks guidance and explanation or assessment for establishing a decision to understand or determine which one is providing an advantage.
a.      Guide and explain the user which algorithm to use, providing some inputs in the "Integration" step, with comparisons against other datasets already used for this, such as* [*https://doi.org/10.1016/j.cell.2021.04.048*](https://urldefense.com/v3/__https:/doi.org/10.1016/j.cell.2021.04.048__;!!NLFGqXoFfo8MMQ!tjS01NNoQ2jc8U-2WS2D1zndcnQfncjvDLMYWMod2el0Tpxq6LokxQu-7l8CxGfWNu4ckffjsAOKwUD9-LOswTd4BpbZh80FUzpYVQ$)*.
b.      Comparing against already published methods and show the benefits of the selected algorithms.*

In our manuscript we now refer to a full set of prior key benchmarking papers that have supported the choice of algorithms incorporated in Panpipes (e.g. for multimodal integration: Luecken *et al.* 2022 *Nature Methods*, Brombacher *et al.* 2022 *Frontiers in Molecular Biosciences,* Lee *et al.* 2023 *bioRxiv*, Makrodimitris *et al*. 2023 *bioRxiv*). We would also like to note that whilst benchmarking studies provide important guidance, no single algorithm will necessarily generate the best results for all datasets, and single-cell integration benchmarking studies themselves can reach different conclusions (e.g. Tran *et al.* 2020 *Genome Biology*, Luecken *et al.* 2022 *Nature Methods*). Hence, Panpipes critically provides users with the flexibility to test and evaluate different algorithms, empowering users to make informed choices. Moreover, rather than needing to run analyses serially so as to test algorithms, which is the case for frameworks (such as Seurat), Panpipes parallelises these runs, thereby saving time and enabling comparisons.

However, given the reviewer’s suggestion we have also improved the documentation on how to run different integration methods, particularly with respect to multimodal datasets, and we have provided guidance on how to interpret the results of the integration using the dataset indicated by the reviewer. Using this dataset, which we also used for providing a biological context for Panpipes’ reference mapping capacity, we provide the biological context for the interpretation of integration results (comparing WNN, totalVI and MOFA), as noted in the relevant results section and through the corresponding UMAP plots generated. These plots have been added to the supplement (Figure S2), given the editorial note that the manuscript is under consideration as a Software article.

**Minor**
1)       *Figure 1 may confuse readers as it shows convergence of all modalities in the Integrated MuData with kNN graph, while the pipeline allows for unique integration methods for each modality combination.*

We thank the reviewer for bringing this to our attention; we have modified Figure 1 accordingly so as to avoid any confusion.

2)       *The responsibility for pipeline maintenance and updates is not discussed.*

We have now included a statement in the manuscript under the “Availability of data and materials” section and in the accompanying documentation, indicating that pipeline maintenance and updates are the responsibility of the co-senior authors and co-first author Fabiola Curion. In addition, Panpipes was created with the view of including it within scverse ecosystem, whose mission is to ensure long-term maintenance of core omics data tools for the life sciences.

3)       *Arguments like GEX or RNA in the yaml file could create confusion and require further description.*

To help avoid confusion, we have modified the yaml file and throughout the manuscript and all associated documentation we should only refer to “RNA” rather than “GEX”, which should not require further clarification.

**Reviewer #2:** *Panpipes is a wonderful and timely tool that provide common workflows for single-cell multi-omics data analysis. The workflows are carefully selected, with the overall framework comprehensively designed. Overall, this collection of workflows will be welcomed by bioinformaticians.*

We thank the reviewer for appreciating the value and utility of Panpipes and the workflows we have chosen to incorporate into the pipeline.

*The review wonders whether providing more information on the following will help highlight the strength and usefulness of Panpipes and facilitate the use of the tool:*

1) *How good the Panpipes tool follows the FAIR principles? It will be wonderful to summarize key functionalities according to the four FAIR principles.*

We thank the reviewer for this excellent suggestion, which we have now incorporated into our manuscript discussion. Briefly, and given the amendments that we have made to the documentation and codebase, we believe that Panpipes adheres to the FAIR principles as follows:

**- Findable:** With the new documentation and tutorials created, which are all linked to the pipeline GitHub, we are ensuring that Panpipes is easy to find, its purpose is clearly conveyed, and we provide detailed information regarding the pipeline overview, its dependencies, and how to implement and use it. Throughout our documentation we also use appropriate tags, labels and keywords to enhance Panpipes searchability. In addition, part of our motivation for submitting this manuscript for publication is to ensure broader exposure and the generation of a digital object identifier, thereby providing a permanent link to the pipeline.

**- Accessible:** To ensure accessibility, Panpipes has been developed with entirely open-access software, the code is available on GitHub (<https://github.com/DendrouLab/panpipes>) and further documentation and tutorials (<https://panpipes-pipelines.readthedocs.io/en/latest/>) are provided to encourage users to adapt the pipeline to their own needs. In addition, Panpipes can now be installed as a PyPi package and its use of conda environments helps to ensure reproducibility.

**- Interoperable:** Panpipes was designed so that all the tools in the scverse ecosystem can interact with the pipeline outputs. We generate MuData, tabular formats and a range of different plots. We also enable data conversion for user-friendly visualisation with the Chan Zuckerberg Initiative’s CELLxGENE browser. Moreover, we chose to design Panpipes with scverse at its core as scverse is interoperable with Bioconductor (R ecosystem).

**- Reusable:** The Panpipes design is modular such that individual modules could potentially be used in other contexts, and the code and pipeline implementation and application are now well-documented to facilitate utility and re-use. The code is available through GitHub to ensure version control, and data provenance (sources of data and transformations performed within the pipeline) are also well-documented.

2) *Data standards. A summary about the current data standards and metadata standards widely used in biomedical consortia of single cell omics data and by funding agencies and how Panpipes supports such standards will be helpful. Especially, starting from this year, NIH requires all proposals and funded projects should follow the new Data Management and Sharing policy. Therefore, many researchers start to examine whether tools are compatible with such requirements. Providing explicit descriptions will help researchers to decide whether they want to use Panpipes.*

As we have now also noted in the main manuscript, Panpipes employs data standards widely used and accepted by the single-cell community. For example, AnnData is a standard data format for the analysis and transfer of single-cell data which has been adopted by the Human Cell Atlas as part of their conventional data formats over the last few years. Moreover, as detailed in the previous point, Panpipes is also FAIR compliant, and hence the pipeline is in line with the new NIH new data management and sharing policy.

3) *Flexibilities of switching specific functionalities to different tools. Panpipes claims that incorporating new omics types is supported, due to the modularity of the system. Meanwhile, since there are so many new tools emerging, just wonder whether authors can describe what interface and design allow Panpipes to adapt new tools, and to specify which tools can be easily customized to replace the current Panpipes tools by the end users, and which needs significant amount of coding. This is particularly important since some choices of tools by Panpipes are different from what my lab and my peers use. I fully respect Panpipes' choices of tools. Meanwhile, whether bioinformaticians can easily use Panpipes to cover tools that we regularly use is a major determinant of whether my lab will use Panpipes.*

Panpipes is modular to allow users to customize their experience, and to enable this Panpipes is written in Python, which is the language widely used by bioinformatics and machine learning analysts. The software implemented in Panpipes, the workflow management language (cgat-core) and Panpipes itself are all now well-documented and all open-software. The code is open access and we welcome submissions from users that who may, for example, wish to add further uni- or multimodal integration methods to the existing tools. Our new documentation includes guidance on how users can best contribute to Panpipes.

4) *The support to spatial omics data. Many single-cell omics tools have recently released their spatial omics pipelines. Here are some examples: 1) Seurat pipeline was not initially developed for spot-level ST data, but for single-cell RNA-Seq data. They have officially claimed that their pipeline can be used for both spot-level ST data (*[*https://satijalab.org/seurat/articles/spatial_vignette.html*](https://urldefense.com/v3/__https:/satijalab.org/seurat/articles/spatial_vignette.html__;!!NLFGqXoFfo8MMQ!tjS01NNoQ2jc8U-2WS2D1zndcnQfncjvDLMYWMod2el0Tpxq6LokxQu-7l8CxGfWNu4ckffjsAOKwUD9-LOswTd4BpbZh81kkJAlVA$)*) and the single-cell ST data (*[*https://satijalab.org/seurat/articles/spatial_vignette_2.html*](https://urldefense.com/v3/__https:/satijalab.org/seurat/articles/spatial_vignette_2.html__;!!NLFGqXoFfo8MMQ!tjS01NNoQ2jc8U-2WS2D1zndcnQfncjvDLMYWMod2el0Tpxq6LokxQu-7l8CxGfWNu4ckffjsAOKwUD9-LOswTd4BpbZh80w7Rxplw$)*). Scanpy also released the official example for single cell ST data (MERFISH data,* [*https://scanpy-tutorials.readthedocs.io/en/latest/spatial/basic-analysis.html#MERFISH-example)*](https://urldefense.com/v3/__https:/scanpy-tutorials.readthedocs.io/en/latest/spatial/basic-analysis.html*MERFISH-example)__;Iw!!NLFGqXoFfo8MMQ!tjS01NNoQ2jc8U-2WS2D1zndcnQfncjvDLMYWMod2el0Tpxq6LokxQu-7l8CxGfWNu4ckffjsAOKwUD9-LOswTd4BpbZh83NgHwlcQ$)*. Just wonder whether Panpipes considers including such supports, or would like to leave it to future versions? Extending supports to spatial omics data can be achieved in a short time period (i.e., during revision time). It will significantly increase the impact of this useful tool.*

We were initially going to release workflows supporting spatial omics data analysis in the next Panpipes release, but as we agree with the reviewer that extending support to spatial data would significantly increase the impact of Panpipes, we have now added relevant workflows, as detailed in the manuscript and the accompanying documentation. These workflows will support the QC, preprocessing and clustering of spatial omics data, including those generated through the 10x Genomics’ Visium and Vizgen’s MERSCOPE platforms. In addition, for spot-level data we also provide a deconvolution workflow employing cell2location (Kleshchevnikov *et al*. 2022 *Nature Biotechnology*) that can make use of external single-cell data or data analysed through the Panpipes single-cell workflows.

5) *Panpipes provides amazing visualization functions and other functions. However, this capacity has not been fully demonstrated in the github documentations. The reviewer strongly recommends including a series of vignettes in github to demonstrate the visualization functionalities as well as how to use Panpipes in other common use cases. The reviewer, and maybe other peers, are so "spoiled" by the decent documentations provided with other bioinformatics tools, and thus expect to have ready-to-use vignettes to start with when using Panpipes in our research. For example, the scanpy tutorials (*[*https://scanpy.readthedocs.io/en/stable/tutorials.html*](https://urldefense.com/v3/__https:/scanpy.readthedocs.io/en/stable/tutorials.html__;!!NLFGqXoFfo8MMQ!tjS01NNoQ2jc8U-2WS2D1zndcnQfncjvDLMYWMod2el0Tpxq6LokxQu-7l8CxGfWNu4ckffjsAOKwUD9-LOswTd4BpbZh83MxYL5gw$)*) are so helpful for my students and colleagues to follow. Could Panpipes consider providing tutorials and vignettes at this level and degree please?*

We thank the reviewer for recognising the quality of our visualisation and other functions provided through Panpipes. We agree that added documentation would be highly helpful for users. We have now created step-by-step installation and usage guidelines and tutorials available through <https://panpipes-pipelines.readthedocs.io/en/latest/> and linked to <https://github.com/DendrouLab/panpipes>, that cover all the individual Panpipes workflows in detail.

**Second round of review**

**Reviewer 1**

We have thoroughly reviewed the revisions made by the authors in response to our comments. The updated manuscript incorporates several significant changes, particularly in the handling of spatial transcriptomics data. As a result, the manuscript is now much clearer and effectively communicates the pipeline's purpose and practicality.
We dedicated a day to installing the tool, running the tutorial pipeline on test data, and attempting to execute it on an in-house dataset comprising ATAC and RNA data. The authors have put in commendable efforts to provide detailed tutorials for using the pipeline, including a PyPi installation method, which worked flawlessly.
In short, we were able to execute the tutorial pipeline without encountering errors. However, it's important to note that we had to address numerous issues along the way. We hope that the authors will rectify these problems before finalizing the paper. Additionally, other users may identify and report these issues on GitHub. While we did not test every aspect, we encourage the authors to do so, as rigorous testing is as crucial as development itself. Here are some major points we encountered (though not an exhaustive list):
• The 'panpipes install_r_dependencies' command did not function correctly, necessitating manual retrieval and execution of the .R install source file from GitHub.
• Numerous typos and errors, such as inconsistencies in capitalization or path errors, appeared in the tutorial pipeline. We had to make corrections during its execution. Proofreading the tutorial and providing example files would be beneficial.
• Certain parameterization options, like using 'dsb' instead of 'clr' normalization, did not work and resulted in failures.

In conclusion, the documentation has improved compared to the previous version, but users may still need to invest a significant amount of time to get the pipeline working or customize it to their specific needs. Some information is currently missing, and we hope the authors will address these issues, especially given their active involvement on GitHub.

We also attempted to run the pipeline on in-house-generated ATAC/RNA 10x multi-omics data but encountered difficulties during the 'ingest' phase. Although it initially appeared straightforward to ingest multimodal data from our 'outs' output folder from Cell Ranger, we found a lack of clear instructions on how to do this correctly. The tutorial offers different options, but they do not seem to work seamlessly, leaving users in a trial-and-error situation, correcting errors as they arise. Configuring the sample file to include various components of the multimodal dataset is unclear, and the order of columns appears to affect pipeline behavior.

Furthermore, our attempts to obtain the 'ten_x_metrics' mentioned in the pipeline were unsuccessful. It is possible that we were missing specific files required for this step, but these were not adequately detailed in the workflow or tutorial.
After configuring the sample file, we faced another challenge in creating a 'pipeline.yml' configuration that would not fail. In our case, the multimodal data was from mice, while the gene list provided by the authors was for humans. Although we created a mouse-specific file, only the ATAC data was processed by the pipeline. After encountering this issue, we decided to discontinue our efforts to use the pipeline.

In conclusion, the pipeline appears to be a significant effort in consolidating various tools for multi-omics and spatial transcriptomics data analysis (although we did not test the latter). However, our concerns about its usability persist, particularly in terms of configuration, which remains quite cumbersome. We believe that the pipeline is not yet fully mature, given the numerous errors we encountered, each of which dampened our enthusiasm and interest. While we see potential applications in bioinformatics facilities or services, we have reservations about labs using it for their own analyses due to the current challenges in configuration and execution. Nonetheless, we remain optimistic that these issues can be resolved over time, but it is evident that the pipeline is still not fully mature at this point.

**Reviewer 2**

The authors have decently addressed most of my concerns.


However, there are still two concerns:

1) some links of Panpipe online materials do not work. For example, when I followed the Tutorials on Spatial Analysis and selected "Deconvoluting spatial data", it led me to the following webpage, which did not work and gave a "404 Not Found" error. https://panpipes-tutorials.readthedocs.io/en/latest/filtering_spatial_data/deconvoluting_spatial_data_with_panpipes.html

Same for the tutorial on "Clustering pipeline"

https://panpipes-tutorials.readthedocs.io/en/latest/clustering/clustering.html

Could the authors double check their online resources please?

2) lack of tutorials for visualization. Among all Panpipes tutorials and documentations the reviewer checked, only one visualization plot is provided ("Assessing background" at https://panpipes-pipelines.readthedocs.io/en/latest/workflows/qc.html#assessing-background). Could the authors provide tutorials with example codes and example plots please?

**Authors Response**

**Point-by-point responses to the reviewers’ comments:**

GBIO-D-23-00361R1 “Panpipes: a pipeline for multiomic single-cell and spatial transcriptomic data analysis”

Reviewer Rebuttal

Reviewer #1: We have thoroughly reviewed the revisions made by the authors in response to our comments. The updated manuscript incorporates several significant changes, particularly in the handling of spatial transcriptomics data. As a result, the manuscript is now much clearer and effectively communicates the pipeline's purpose and practicality.

We dedicated a day to installing the tool, running the tutorial pipeline on test data, and attempting to execute it on an in-house dataset comprising ATAC and RNA data. The authors have put in commendable efforts to provide detailed tutorials for using the pipeline, including a PyPi installation method, which worked flawlessly.

In short, we were able to execute the tutorial pipeline without encountering errors.

>> We would like to take this opportunity to thank the reviewer for their thorough review and constructive feedback on our manuscript. We are happy to learn that the revisions and improvements we made, especially in the handling of the installation of panpipes, have enhanced the clarity and practicality of our work.

1) However, it's important to note that we had to address numerous issues along the way. We hope that the authors will rectify these problems before finalizing the paper. Additionally, other users may identify and report these issues on GitHub. While we did not test every aspect, we encourage the authors to do so, as rigorous testing is as crucial as development itself. Here are some major points we encountered (though not an exhaustive list):
• The 'panpipes install_r_dependencies' command did not function correctly, necessitating manual retrieval and execution of the .R install source file from GitHub.
• Numerous typos and errors, such as inconsistencies in capitalization or path errors, appeared in the tutorial pipeline. We had to make corrections during its execution. Proofreading the tutorial and providing example files would be beneficial.
• Certain parameterization options, like using 'dsb' instead of 'clr' normalization, did not work and resulted in failures.
In conclusion, the documentation has improved compared to the previous version, but users may still need to invest a significant amount of time to get the pipeline working or customize it to their specific needs. Some information is currently missing, and we hope the authors will address these issues, especially given their active involvement on GitHub.

>> We thank the reviewer for the detailed feedback and for identifying specific areas of concern in our pipeline. We sincerely appreciate the time and effort they have invested in thoroughly testing our pipeline and providing these valuable insights.

Installation issues:
We apologize for the inconvenience caused by the malfunction of the 'panpipes install_r_dependencies' command. Based on your feedback, we have reviewed and revised the installation process. The updated version now includes a more reliable method for installing R dependencies, directly addressing the issue encountered. We have also added more details to the documentation to help users troubleshoot R installation.

Tutorial accuracy:
We acknowledge the importance of clear and error-free tutorials for user comprehension and experience. Following your observation regarding typos and inconsistencies, we have comprehensively proof-read and amended our tutorial documentation. We have also uploaded to figshare any file used in our tutorial and/or clearly referenced the source data when using publicly available datasets.

Parameterisation options:
We thank the reviewer for their feedback on the two protein normalisation methods supported in Panpipes. To be sure to facilitate a smoother execution process for future users, we have reviewed our code and have included a new tutorial demonstrating the normalisation using CITE-Seq data (RNA + Protein) from raw inputs (https://panpipes-tutorials.readthedocs.io/ en/latest/ingesting_multimodal_data/ingesting_multimodal_data.html), which should clarify the use of the appropriate normalisation for the protein modality when raw inputs are available. Furthermore, we have added more documentation on the normalisation methods supported (https://panpipes-pipelines.readthedocs.io/en/latest/usage/ normalization_methods.html#) and we rectified the configuration files for misleading information.

2) We also attempted to run the pipeline on in-house-generated ATAC/RNA 10x multi-omics data but encountered difficulties during the 'ingest' phase. Although it initially appeared straightforward to ingest multimodal data from our 'outs' output folder from Cell Ranger, we found a lack of clear instructions on how to do this correctly. The tutorial offers different options, but they do not seem to work seamlessly, leaving users in a trial-and-error situation, correcting errors as they arise. Configuring the sample file to include various components of the multimodal dataset is unclear, and the order of columns appears to affect pipeline behavior.

Furthermore, our attempts to obtain the 'ten_x_metrics' mentioned in the pipeline were unsuccessful. It is possible that we were missing specific files required for this step, but these were not adequately detailed in the workflow or tutorial. After configuring the sample file, we faced another challenge in creating a 'pipeline.yml' configuration that would not fail. In our case, the multimodal data was from mice, while the gene list provided by the authors was for humans. Although we created a mouse-specific file, only the ATAC data was processed by the pipeline. After encountering this issue, we decided to discontinue our efforts to use the pipeline.

>> Based on the reviewer’s feedback, we have thoroughly reviewed the code and as a result we have included a new tutorial on the ingestion of multiome data from raw inputs (https://panpipes-tutorials.readthedocs.io/en/latest/ingesting_multiome/ ingesting_mome.html), we have improved the documentation on generating sample submission files ( https://panpipes-pipelines.readthedocs.io/en/latest/usage/ setup_for_qc_mm.html#supported-input-filetypes) and on creating and using custom genes files (https://panpipes-pipelines.readthedocs.io/en/latest/usage/gene_list_format.html), and we have also included a tutorial on ingesting mouse data (https://panpipes-tutorials. readthedocs.io/en/latest/ingesting_mouse/Ingesting_mouse_data_with_panpipes.html), which we believe our users will benefit from.

Furthermore, the ten_x_metrics task is now exemplified in two tutorials (https://panpipes-tutorials.readthedocs.io/en/latest/ingesting_multiome/ingesting_mome.html for the multiome dataset, and https://panpipes-tutorials.readthedocs.io/en/latest/ingesting_multimodal_data/ ingesting_multimodal_data.html for the CITE-Seq dataset).

3) In conclusion, the pipeline appears to be a significant effort in consolidating various tools for multi-omics and spatial transcriptomics data analysis (although we did not test the latter). However, our concerns about its usability persist, particularly in terms of configuration, which remains quite cumbersome. We believe that the pipeline is not yet fully mature, given the numerous errors we encountered, each of which dampened our enthusiasm and interest. While we see potential applications in bioinformatics facilities or services, we have reservations about labs using it for their own analyses due to the current challenges in configuration and execution. Nonetheless, we remain optimistic that these issues can be resolved over time, but it is evident that the pipeline is still not fully mature at this point.

>> As we believe that clear documentation is the cornerstone of an effective and user-friendly pipeline, we have carefully taken on board all reviewer comments to make the necessary improvements to ensure that the pipeline is now ready for use in its intended context.

We have dedicated all efforts to addressing the errors and improving the configuration process to make it as user-friendly as possible. The pipeline has been rigorously tested and refined, particularly in response to the feedback received from users on our GitHub page - on which we are actively engaged as the reviewer pointed out. We are confident that the current version is robust and capable of performing the necessary tasks effectively. We are also committed to the ongoing support and continuous improvement of the pipeline and its documentation to ensure we increase our user base.


Reviewer #2: The authors have decently addressed most of my concerns.

However, there are still two concerns:

1) some links of Panpipe online materials do not work. For example, when I followed the Tutorials on Spatial Analysis and selected "Deconvoluting spatial data", it led me to the following webpage, which did not work and gave a "404 Not Found" error. https://panpipes-tutorials.readthedocs.io/en/latest/filtering_spatial_data/deconvoluting_spatial_data_with_panpipes.html

Same for the tutorial on "Clustering pipeline" https://panpipes-tutorials.readthedocs.io/ en/latest/clustering/clustering.html

Could the authors double check their online resources please?

>> We are grateful for the reviewer’s overall feedback as it helps us improve the user experience for our tool. We thank the reviewer for bringing the issues with our online documentation to our attention. We sincerely apologize for the inconvenience caused by the non-functional links in Panpipes’ online materials. Upon investigation, we discovered that these issues were due to a typo in our automated update system. We have now rectified this error, and we have implemented a more robust review process for all updates to our website. We are pleased to confirm to the reviewer that the links they mentioned, as well as the others, are now functioning correctly.

2) Lack of tutorials for visualization. Among all Panpipes tutorials and documentations the reviewer checked, only one visualization plot is provided ("Assessing background" at https://panpipes-pipelines.readthedocs.io/en/latest/workflows/qc.html#assessing-background). Could the authors provide tutorials with example codes and example plots please?

>> We understand the importance of reliable and accessible online resources, especially for complex tools like ours. Therefore, we greatly appreciate the reviewer’s feedback on the need for more comprehensive visualisation tutorials in Panpipes’ documentation.

We have now included example plots in every tutorial within our documentation. This enhancement will allow users to better understand the expected outputs and how to interpret them effectively.

Additionally, we have developed a dedicated visualisation tutorial that provides detailed example codes and corresponding example plots. We believe these additions will greatly improve the usability and learning curve of our pipeline, ensuring a more intuitive and informative experience. The updated tutorials with integrated visualisation examples can be accessed at https://panpipes-pipelines.readthedocs.io/en/latest/tutorials/index.html.

We believe in the potential of Panpipes to significantly contribute to bioinformatics research. Our pipeline fills a much-needed niche for comparative single-cell multiomics where flexibility for data exploration, speed and scalability are key. We are committed to continually improving the usability of Panpipes and we actively monitor our GitHub page to ensure that all user feedback and suggestion are taken on board and readily implemented.

**Third round of review**

**Reviewer 1**

We appreciate the authors' pipeline updates, which seem to address many of the stipulated concerns. We would like to point out that we spent -again- quite some time testing some aspects of the pipeline, but -due to time constraints- were unable to cover the spatial transcriptomics part.

Specifically:
(1) The setup/installation of the tool is well-documented and functional.
(2) The ingestion process remains somewhat challenging and time-consuming. Clarification is needed, particularly regarding the placement of markers (mt, rp, hg genes) inside the qc_file or a separate file that the pipeline reads. The configuration of pipeline.yml, including aspects like scoring genes, requires better explanation.
(3) The pipeline.yml includes commented lines that seem outdated from the previous revision. It would be helpful to clarify whether these are to be uncommented based on the type of modalities being worked on.
(4) While the submission_file is better documented, providing data in the required format remains unclear.
(5) Quality check files need better grouping, and instructions on associating score_genes or markers into the file and pipeline.yml are lacking. Are there errors if the column of the qc_file or custom_genes is in uppercase or lowercase?
(6) Some package versions, such as scvi, continually throw errors as they are not updated in the panpipes workflow.
(7) Error messages from the pipeline are extensive and unclear. It would be beneficial to provide clearer error messages for easier troubleshooting.
(8) The preprocessing workflow is straightforward and effective.
(9) The integration step is poorly documented due to the inclusion of various tools. The tutorial does not cover the full range of algorithms, leaving users to figure them out independently.
(10) While we managed to run the unimodal batch corrections with one set of parameters, all other attempts failed.

Despite our efforts over one day to investigate and use the pipeline, its difficulty in use and parameterization led us to abort its use. This aligns with the challenges mentioned by both reviewers, reinforcing our primary conclusion that, while the pipeline consolidates tools for multi-omics and spatial transcriptomics data analysis, concerns about its usability persist. The current configuration challenges may limit its application for labs conducting their own analyses. Our main conclusion remains thus largely unchanged:

"The pipeline represents a significant effort in consolidating various tools for multi-omics and spatial transcriptomics data analysis. However, persistent concerns about usability, especially in terms of configuration, raise doubts about its suitability for labs conducting their analyses. While its potential applications in bioinformatics facilities or services are recognized, we recommend further enhancements to address current challenges in configuration and execution."

**Authors Response:**

**GBIO-D-23-00361R2 “Panpipes: a pipeline for multiomic single-cell and spatial transcriptomic data analysis” – Response to Reviewer Comments**

Reviewer #1

[Reviewer comments are in *green italics*; author responses are in black, with links and code and documentation excerpts in blue.]

*We appreciate the authors' pipeline updates, which seem to address many of the stipulated concerns. We would like to point out that we spent -again- quite some time testing some aspects of the pipeline, but -due to time constraints- were unable to cover the spatial transcriptomics part.*

*Despite our efforts over one day to investigate and use the pipeline, its difficulty in use and parameterization led us to abort its use. This aligns with the challenges mentioned by both reviewers, reinforcing our primary conclusion that, while the pipeline consolidates tools for multi-omics and spatial transcriptomics data analysis, concerns about its usability persist. The current configuration challenges may limit its application for labs conducting their own analyses. Our main conclusion remains thus largely unchanged:*

*"The pipeline represents a significant effort in consolidating various tools for multi-omics and spatial transcriptomics data analysis. However, persistent concerns about usability, especially in terms of configuration, raise doubts about its suitability for labs conducting their analyses. While its potential applications in bioinformatics facilities or services are recognized, we recommend further enhancements to address current challenges in configuration and execution."*

Whilst Reviewer 2 was satisfied with the amendments to the code and documentation of the Panpipes pipeline in the previous revision, this Reviewer had outstanding issues with respect to ease of use. We thank this Reviewer for testing our pipeline and for their detailed feedback on usability, particularly with respect to documentation.

In order to ensure that we could fully address the Reviewer’s comments we put together a focus group of users new to Panpipes who either had prior experience in R or who were new to computational biology entirely, including postdoctoral researchers, PhD students, MSc students and a wet-lab research assistant. Focus group members have been included as co­authors or have been mentioned in our acknowledgements section depending on the degree of contribution. In addition to the focus group, we also brought on board a Bioconductor expert and another pipelining expert – both with biomedical data science training expertise – to ensure we can fully cater to R users.

Based on the Reviewer’s comments and the feedback from the focus group and additional experts, we have made significant changes to the documentation and the code of the pipeline. In overview:

-	We have added new documentation pages to help users familiarise with the scverse ecosystem:

<https://panpipes-pipelines.readthedocs.io/en/latest/usage/panpipes_scverse.html>

-	We have simplified the 9 workflows configuration files

-	We have added documentation files on each workflow configuration parameters at: <https://panpipes-pipelines.readthedocs.io/en/latest/yaml_docs/index.html>

-	We have included documentation pages explaining the formatting and usage of yaml files: <https://panpipes->

[pipelines.readthedocs.io/en/latest/yaml_docs/useful_info_on_yml.html](http://pipelines.readthedocs.io/en/latest/yaml_docs/useful_info_on_yml.html)

-	We have added additional instructions for installation on different operating systems: <https://panpipes-pipelines.readthedocs.io/en/latest/install.html>

-	We have added specific instructions to install panpipes versions for multi-omics and spatial analysis

-	We have systematically improved our logging systems throughout the pipeline

-	We include a troubleshooting page to help debugging errors: <https://panpipes>-pipelines.readthedocs.io/en/latest/usage/troubleshooting.html

-	We have implemented GitHub actions to ensure Continuous Improvement while we maintain and keep developing components: <https://github.com/DendrouLab/panpipes/tree/main/.github/workflows>. GitHub Actions is a powerful automation tool integrated into GitHub, enabling continuous integration and continuous deployment (CI/CD) directly within the repository hosting environment. This feature allows for automated testing of our pipeline each time changes are made, ensuring that new code contributions do not disrupt existing functionalities. The inclusion of these actions reflects our commitment to maintaining a well-tested and stable tool for the analysis of single-cell multimodal and spatial data.

-	We have added documentation on the formatting and usage of gene QC files with additional QC files for mouse references: <https://panpipes-pipelines.readthedocs.io/en/latest/usage/gene_list_format.html>

-	We have added documentation on integration algorithms supported in panpipes: <https://panpipes-pipelines.readthedocs.io/en/latest/usage/integration_methods.html>

-	We have also added a further spatial deconvolution algorithm, Tangram, in the spatial deconvolution workflow: <https://panpipes-pipelines.readthedocs.io/en/latest/workflows/deconvolute_spatial.html#tangram>

-	We have also updated the integration workflow to include the more efficient version of the scib_metrics package (<https://scib-metrics.readthedocs.io/en/stable/)>

Given these changes – many recommended by the Reviewer – we are pleased to note that the Panpipes user base has been steadily expanding and includes:

-	Academic and industry members of the international Oxford-Janssen Cartography Consortium (that spans 15 different disease areas; <https://www.medsci.ox.ac.uk/for-staff/resources/business-partnerships-office/research-alliances/research-alliance-with-janssen/oxford-janssen-working-in-collaboration)>

- The Chan Zuckerberg Initiative-funded international LEGACY Network (<https://www.imperial.ac.uk/news/231957/million-investment-study-investigating-vaccines-work/)>

- The COMBATcancer programme

(<https://www.cancer.ox.ac.uk/research/projects/combatcancer)>

- The international inflammatory bowel disease therapeutic single-cell atlas studies TAURUS (<https://www.biorxiv.org/content/10.1101/2023.05.05.539635v1)> and SAGITTARIUS (<https://www.kennedy.ox.ac.uk/news/new-research-endoscopy-service-launches-today)>

- The single-cell asthma drug discovery study, MORSE

(<https://www.proactiveinvestors.co.uk/companies/news/957265/sensyne-health-teams-up-with-oxford-university-to-find-new-treatments-for-asthma-957265.html)>

-	Further users at the Institute of Computational Biology-Helmholtz Munich in

Germany, University College London and the University of Cambridge in the UK, and KULeuven in Belgium, to name a few.

Training workshops have also been run at the Centre for Human Genetics in Oxford and in European Bioinformatic summer schools, for example via the NGSchool (<https://ngschool.eu/),> with more planned through the Oxford Biomedical Data Science Training Programme (<https://www.imm.ox.ac.uk/research/units-and-centres/mrc-wimm-centre-for-computational-biology/training/oxford-biomedical-data-science-training-programme),> for example.

Lastly, we are excited that Panpipes has just been approved as one of the packages to be included in the scverse ecosystem (<https://github.com/scverse/ecosystem-packages).>

(1) *The setup/installation of the tool is well-documented and functional.*

We thank the reviewer for acknowledging that the setup is functional. During our documentation re-haul we have also added more instructions for different Operating Systems. Detailed instructions are available at: <https://panpipes-pipelines.readthedocs.io/en/latest/install.html>

(2) *The ingestion process remains somewhat challenging and time-consuming. Clarification is needed, particularly regarding the placement of markers (mt, rp, hg genes) inside the qc_file or a separate file that the pipeline reads. The configuration of pipeline.yml, including aspects like scoring genes, requires better explanation.*

We thank the Reviewer for their feedback on the ingestion process. To address these concerns, we have significantly enhanced our documentation and tutorials, aiming to simplify and accelerate the setup and operational phases. Moreover, in this round of revision, we convened a focus group comprising users with varying levels of expertise in single-cell analysis in both R and Python (including computational novices and R and pipelining experts), to evaluate and provide feedback on our ingestion workflow. Their valuable insights have led to targeted improvements that we believe have both simplified the process and reduced the time required for data ingestion. We are committed to ongoing refinement and are confident that these enhancements will continue to improve user experience and workflow efficiency.

In particular, we have implemented the following changes:

1. We provide documentation on configuring the ingestion pipeline:

<https://panpipes->

[pipelines.readthedocs.io/en/latest/yaml_docs/pipeline_ingestion_yml.html](http://pipelines.readthedocs.io/en/latest/yaml_docs/pipeline_ingestion_yml.html)

2. We provide 6 tutorials covering different ingestion scenarios:

i. <https://panpipes->

[tutorials.readthedocs.io/en/latest/ingesting_data/Ingesting_data_with_panpipes.html](http://tutorials.readthedocs.io/en/latest/ingesting_data/Ingesting_data_with_panpipes.html)

ii. <https://panpipes->

[tutorials.readthedocs.io/en/latest/ingesting_visium_data/Ingesting_visium_data_with_](http://tutorials.readthedocs.io/en/latest/ingesting_visium_data/Ingesting_visium_data_with_) panpipes.html

iii. <https://panpipes->

[tutorials.readthedocs.io/en/latest/ingesting_merfish_data/Ingesting_merfish_data_wit](http://tutorials.readthedocs.io/en/latest/ingesting_merfish_data/Ingesting_merfish_data_wit) h_panpipes.html

iv. <https://panpipes->

[tutorials.readthedocs.io/en/latest/ingesting_multiome/ingesting_mome.html](http://tutorials.readthedocs.io/en/latest/ingesting_multiome/ingesting_mome.html)

v. <https://panpipes->

[tutorials.readthedocs.io/en/latest/ingesting_multimodal_data/ingesting_multimodal_d](http://tutorials.readthedocs.io/en/latest/ingesting_multimodal_data/ingesting_multimodal_d) ata.html

vi. <https://panpipes->

[tutorials.readthedocs.io/en/latest/ingesting_mouse/Ingesting_mouse_data_with_panp](http://tutorials.readthedocs.io/en/latest/ingesting_mouse/Ingesting_mouse_data_with_panp) ipes.html

3. We use preformatted gene lists to calculate QC metrics in the ingestion workflow. We provide examples of these lists:

i. Human identifiers qc gene list:

<https://github.com/DendrouLab/panpipes/blob/main/panpipes/resources/qc_genelist_> 1.0.csv

ii. Human identifiers cell cycle list:

<https://github.com/DendrouLab/panpipes/blob/main/panpipes/resources/cell_cycle_g> enes.tsv

iii. Mouse identifiers qc gene list:

<https://github.com/DendrouLab/panpipes/blob/main/panpipes/resources/qc_gene_list> mouse.csv

iv. Mouse identifiers cell cycle list:

<https://github.com/DendrouLab/panpipes/blob/main/panpipes/resources/mouse_cell_> cycle_genes.tsv

We allow full customization of the gene lists to avoid bottlenecks when processing data, for example from multiple species. We have updated our documentation to clarify how Panpipes uses different QC files and how to format them. We explain how to create, provide and use the qc files here: <https://panpipes-pipelines.readthedocs.io/en/latest/usage/gene_list_format.html>

and we include an excerpt of the documentation here:

[...]

**Custom gene list**

We provide an example of a preformatted gene lists file in

<https://github.com/DendrouLab/panpipes/blob/main/panpipes/resources/qc_genelist_1.0.csv>

All Custom Gene Lists files provided to the pipeline should be in a 3 columns format, where the column headers are "mod" (modality: "rna", "prot", or "atac"), feature and group.

The group column is used to label and distinguish different gene groups, in the same way as you would submit them as a single vector. The same gene can belong to multiple groups by duplicating the row for that gene and changing the group label.

Gene ids can come in a variety of formats, including upper or lower cases, letters, numbers or a combination of both.

Users can provide any gene format in the custom gene files, depending on the reference they used to produce their count matrices.

- **mod**: the modality for the feature in use. Modalities are always specified in lowercase.

- **feature**: feature name, i.e. a gene id

- **group**: the group the gene belongs to. Group can be upper or lowercase or a mix of both

and will be interpreted as a string.

| mod | feature | group |
| --- | --- | --- |
| rna | gene_1 | mt |
| rna | gene_2 | rp |
| rna | gene_1 | exclude |
| rna | gene_1 | markerX |
| ... | ... | ... |

For example, if your count matrix uses Ensembl ids, you would specify `ENSG00000163914` in the feature column and group `photoreceptor`

```

GeneCards Symbol: RHO

HGNC: 10012

NCBI Gene: 6010

Ensembl: ENSG00000163914

**The gene lists are not pre-determined within panpipes in order to maximise flexibility and users can provide their own lists.**

[...]

[...]

**Using custom gene lists to calculate QC metrics**

Panpipes uses "actions" to define in which tasks to use the provided gene list. We encode three main actions to use gene lists to describe qualities of the cells and populate the metadata (`.obs`) of the object with the newly calculated cell QC metric. Specify the "group" name of the genes you want to use to apply a specific action to calculate the cell QC metric

If left blank, these actions will not be performed (i.e. no calculation of % of mt genes per cell will be included in the ingestion of the data)

If the group name is not spelled correctly, the action will fail. See [The section on] (#explaining-custom-gene-lists-actions)

**Supplying custom gene lists to calculate QC metrics**

The human custom genelist file can be supplied by the user in the workflows configuration files:

1. **Ingest workflow**

pipeline_ingest config file: (pipeline.yml)

Supply the gene list by customizing the following parameter

```

custom_genes_file: resources/qc_genelist_1.0.csv

```

2. **Preprocess workflow**

pipeline_preprocess config file: (pipeline.yml)

Supply the gene list by customizing the following parameter

```

exclude_file: resources/qc_genelist_1.0.csv

```

*Note that we have formatted an example file containing all genes to use in both workflows, and therefore supply the same file to both workflows but users can have independent files for each of them.*

If the input is from mouse data

**2. Ingest workflow**

pipeline_ingest config file: (pipeline.yml)

```

custom_genes_file:resources/qc_gene_list_mouse.csv ```

2. **Preprocess workflow**

pipeline_preprocess config file: (pipeline.yml)

```

exclude_file: resources/qc_gene_list_mouse.csv

```

**Explaining custom gene lists actions**

**1. Ingest workflow** (pipeline_ingest.py)

In the qc section of the `ingest` workflow, we specify the main actions harnessing the gene list provided in the `custom_genes_file` param:

```

custom_genes_file: resources/qc_genelist_1.0.csv

calc_proportions: hb,mt,rp

score_genes: mt

ccgenes: default

```

**- calc_proportions:** calculate proportion of reads mapping to X genes over total number of reads, per cell, using scanpy.pp.calculate_qc_metrics (<https://scanpy.readthedocs.io/en/stable/generated/scanpy.pp.calculate_qc_metrics.html#sc> anpy.pp.calculate_qc_metrics).

For example, for the rna modality, including a list of mitochondrial genes in the group `mt`, and setting

calc_proportion: mt

will calculate the proportion of reads mapping to the genes whose group is "mt" and and will add `pct_counts_mt`, and `total_counts_mt` to the `mdata["rna"].obs` assay.

**- score_genes:** using scanpy.tl.score_genes

(<https://scanpy.readthedocs.io/en/stable/generated/scanpy.tl.score_genes.html),> the action calculates the average expression of a set of genes, subtracted of the average expression of a reference set of genes. First introduced in Satija et al. Nature Biotechnology (2015).

This action will generate a column **GroupName_score** and add it to the `mdata[MOD].obs` assay. For example for the rna modality, including a list of Markers for a cell type in the group 'MarkersNeutro' and setting

score_genes: MarkersNeutro

will score the cells for the list of genes provided and add a column `MarkersNeutro_score` to the `mdata["rna"].obs` assay.

**2. Preprocess workflow** (pipeline_preprocess.py)

In the preprocess workflow, we specify what action to perform on the genes in the custom gene list using the `exclude` parameter.

```

exclude_file: resources/qc_genelist_1.0.csv

exclude: default

```

**- exclude:** exclude these genes from the HVG selection, if they are deemed Highly Variable.

For the exclude action, if set to `default` the workflow will look for genes whose group is set to `exclude` in the supplied qc_genelist file. Alternatively, if you are specifying your custom gene list and you want to exclude another set of genes, for example a group you call `TCR_genes`, specify this group (i.e. `exclude: TCR_genes`) If left blank, no genes will be excluded from the HVG.

**Cell cycle actions**

As described before, we also rely on a user-supplied list of genes to calculate the cell cycle phase of a cell. We believe that this choice offers the maximum flexibility to use a trusted gene-set for the calculation of this metric.

The cell cycle scoring happens in the `ingest` workflow using the `ccgenes` parameter. The cell cycle action is performed using [`scanpy.tl.score_genes_cell_cycle`](<https://scanpy.readthedocs.io/en/stable/generated/scan> py.tl.score_genes_cell_cycle.html)

```

ccgenes: default

```

**ccgenes:**

Setting the `ccgenes` param to `default` in the ingest workflow will calculate the phase of the

cell cycle in which the cell is by using `scanpy.tl.score_genes_cell_cycle` using the file

provided in `panpipes/resources/cell_cicle_genes.tsv`. Using this file, this action will produce

at least 3 columns in the `mdata["rna"].obs` assay, namely 'S_score', 'G2M_score', 'phase'.

Users can create their own list, and need to specify the path to this new file in the `ccgenes`

param to score the cells with their custom list.

If left blank, the cellcycle score won't be calculated.

[...]

4. We have also simplified the ingest workflow configuration file which now groups the quality check parameters all in the same place. Below is an excerpt of the INGEST configuration yaml:

**```**

```
#
# RNA modality Quality Control
# Providing a gene list
# see documentation at https://panpipes-
pipelines.readthedocs.io/en/latest/usage/gene_list_format.html custom_genes_file: resources/qc_genelist_1.0.csv
# Defining actions on the genes
# (for pipeline_ingest.py)
calc_proportions: hb,mt,rp
score_genes:
# cell cycle action
ccgenes: default
```

**```**

All of our example files are available to inspect at:

<https://github.com/DendrouLab/panpipes/tree/main/panpipes/resources>

and the relevant files are referenced throughout our documentation.

*(3) The pipeline.yml includes commented lines that seem outdated from the previous revision. It would be helpful to clarify whether these are to be uncommented based on the type of modalities being worked on.*

Panpipes parses input data and parameters by interacting with a set of configuration files, one for each workflow, which are written in the YAML format. In the YAML format, commented lines are ignored when the code reads the file. Originally, we included instructions and descriptions of each parameter within each configuration file, but we appreciate this choice may render the configuration files long and difficult to understand, and may confuse the user, specifically regarding which parameters are parsed when running the workflow.

Therefore, we implemented the following changes:

1. We have added documentation explaining details about the YAML format: <https://panpipes-pipelines.readthedocs.io/en/latest/yaml_docs/useful_info_on_yml.html>
2. We have simplified all the configuration files the 9 workflows included in panpipes, in particular the INGESTION configuration yaml can be inspected at: <https://github.com/DendrouLab/panpipes/blob/main/panpipes/panpipes/pipeline_inge> st/pipeline.yml
3. We provide detailed documentation pages on the 9 workflows implemented in panpipes
i. INGESTION <https://panpipes->

[pipelines.readthedocs.io/en/latest/yaml_docs/pipeline_ingestion_yml.html](http://pipelines.readthedocs.io/en/latest/yaml_docs/pipeline_ingestion_yml.html)

ii. PREPROCESS <https://panpipes->

[pipelines.readthedocs.io/en/latest/yaml_docs/pipeline_preprocess_yml.html](http://pipelines.readthedocs.io/en/latest/yaml_docs/pipeline_preprocess_yml.html)

iii. INTEGRATION <https://panpipes->

[pipelines.readthedocs.io/en/latest/yaml_docs/pipeline_integration_yml.html](http://pipelines.readthedocs.io/en/latest/yaml_docs/pipeline_integration_yml.html)

iv. CLUSTERING <https://panpipes->

[pipelines.readthedocs.io/en/latest/yaml_docs/pipeline_clustering_yml.html](http://pipelines.readthedocs.io/en/latest/yaml_docs/pipeline_clustering_yml.html)

v. VISUALIZATION <https://panpipes->

[pipelines.readthedocs.io/en/latest/yaml_docs/pipeline_visualization_yml.html](http://pipelines.readthedocs.io/en/latest/yaml_docs/pipeline_visualization_yml.html)

vi. REFMAP <https://panpipes->

[pipelines.readthedocs.io/en/latest/yaml_docs/pipeline_refmap_yml.html](http://pipelines.readthedocs.io/en/latest/yaml_docs/pipeline_refmap_yml.html)

vii. SPATIAL INGESTION <https://panpipes->

[pipelines.readthedocs.io/en/latest/yaml_docs/spatial_qc.html](http://pipelines.readthedocs.io/en/latest/yaml_docs/spatial_qc.html)

viii. SPATIAL PREPROCESSING <https://panpipes->

[pipelines.readthedocs.io/en/latest/yaml_docs/spatial_preprocess.html](http://pipelines.readthedocs.io/en/latest/yaml_docs/spatial_preprocess.html)

ix. SPATIAL DECONVOLUTION <https://panpipes->

[pipelines.readthedocs.io/en/latest/yaml_docs/spatial_deconvolution.html](http://pipelines.readthedocs.io/en/latest/yaml_docs/spatial_deconvolution.html)

*(4) While the submission_file is better documented, providing data in the required format remains unclear.*

We now provide additional documentation on the types of files and required formats. In particular, for the documentation of the ingestion setup this is detailed here: <https://panpipes-pipelines.readthedocs.io/en/latest/usage/setup_for_qc_mm.html>

We include an excerpt of the documentation here:
[...]

The multimodal quality control pipeline (ingest) requires a sample submission file which is used to ingest the data from various formats into a MuData multimodal container that the pipeline can interact with. The sample submission file is a tab-separated file with a minimum of 3 required columns, and one sample per row

The minimum required columns are

| sample_id | rna_path | rna_filetype |
| --- | --- | --- |

If you want to ingest multiple modalities, you need to add additional columns to the input file. The order of the columns is irrelevant, but the names are fixed as follow:

rna_path and rna_filetype for rna modality

prot_path and prot_filetype when you have protein data

atac_path and atac_filetype when you have atac data

for the repertoire data

tcr_path and tcr_filetype
bcr_path and bcr_filetype

**Submission file columns**

**sample id:** Each row must have a unique sample ID. It doesn’t necessarily correspond to the folder where the input sample is stored, but will be used from the ingestion onward to identify this sample. For example, in your dataset with data from six individuals, you might have sample names like ‘Sample_1’ to ‘Sample_6,’ but you can choose to name your sample something more meaningful to you, like ‘pbmc_control’,’pbmc_treated’.

**{X}_paths:** If giving a cellranger path, give the path folder containing all the cellranger outputs, known as the outs folder. Otherwise path should be the complete path to the file. If you have cellranger outputs which have rna and prot within the same files, specify the same path in rna_path and prot_path. The same applies to multiome and cellranger multi outputs.

**{X}_filetype:** The “filetype” column tells panpipe how to read in the data. Panpipes supports a range of inputs. See the supported input filetypes below to see the options for the {X}_filetype columns

**Additional input files when processing atac data**

Panpipes supports reading ATAC/multiome data from a single sample. If you have multiple samples you have to merge them before ingesting with panpipes to ensure that the peak coordinates are harmonized across samples, since simple concatenation would induce undesired batch effects. We recommend using cellranger. or cellatac to aggregate the peaks across samples. Check the signac documentation for a detailed explanation of the steps (you can use this approach to merge samples if you don’t have the cellranger outputs)

When available, you can include additional files from the cellranger outputs under the following three columns:

**peak_annotation_file:** file containing peaks mapped to gene based on the genomic location of the nearby gene. This is a tabular format tab separated (usually .tsv). See general 10X info

**per_barcode_metrics_file** a tabular format .csv file containing metrics for every observed barcode. See general 10X info

**fragments_file :** cellranger outputs this large BED-like tabular file, where each line represents a unique ATAC-seq fragment captured by the assay. Normally, the pipeline outs/ folder contains fragments.tsv.gz and a fragments.tsv.gz.tbi index generated with tabix. panpipes expects both there, and it will fail to read the atac modality if you specified a fragment file but the index is missing. You can generate your own index file using tabix.

Note panpipes supports both tab or comma-separated tables.
[...]

[...]

For each modality per sample, specify the value in the key column in the X_filetype columns

| modality | key | description |
| --- | --- | --- |
| rna/prot/atac | cellranger | the “outs” folder produced by cellranger count |
| rna/prot/atac | cellranger_multi | the “outs” folder produced by cellranger multi |
| rna/prot/atac | 10X_h5 | outs/*.h5 file (filtered or raw matrix) produced by cellranger |
| rna/prot/atac | hd5 | Read a generic .h5 (hdf5) file. |
| rna/prot/atac | h5ad | Anndata h5ad objects (one per sample) |
| rna/prot/atac | txt_matrix | tab-delimited file (one per sample) |
| rna/prot/atac | csv_matrix | comma-delimited file (one per sample) |
| tcr/bcr | cellranger_vdj | Path to filtered_contig_annotations.csv, all_contig_annotations.csv or all_contig_annotations.json. produced by cellranger vdj further details |
| tcr/bcr | tracer | data from TraCeR |
| tcr/bcr | bracer | data from BraCeR |

| tcr/bcr | airr | airr formatted tsv |
| --- | --- | --- |

[...]

(5) *Quality check files need better grouping, and instructions on associating score_genes or markers into the file and pipeline.yml are lacking. Are there errors if the column of the qc_file or custom_genes is in uppercase or lowercase?*

We have simplified the ingest workflow configuration file which now groups the quality check parameters all in the same place. Below is an excerpt of the INGEST configuration yaml:

```

```
#
# RNA modality Quality Control
# Providing a gene list
# see documentation at https://panpipes-
pipelines.readthedocs.io/en/latest/usage/gene_list_format.html custom_genes_file: resources/qc_genelist_1.0.csv
# Defining actions on the genes
# (for pipeline_ingest.py)
calc_proportions: hb,mt,rp
score_genes:
# cell cycle action
ccgenes: default
```

```

In response to comment (2), we have also provided a detailed answer on using and customizing qc files, on scoring genes and on capitalization of genes in the qc files, and we thus refer the Reviewer to the reply above for further details.

(6) *Some package versions, such as scvi, continually throw errors as they are not updated in the panpipes workflow.*

As the Reviewer confirmed, our installation was effective (noted in comment (1)), but given this scvi issue we have extensively tested the package and the other packages installed with Panpipes. We wish to point out the tools we support are all under active development, so the failures encountered with scvi were not due to Panpipes per se but to specific issues on that individual package. For example, these are some of the issues that we encountered which we presume are similar to what the Reviewer experienced and are now addressed in our latest release:

-	<https://github.com/scverse/scvi-tools/issues/2582> -* on jax dependencies

-	<https://github.com/scverse/scvi-tools/issues/2581> -* on failures with Multivi on scvi-tools version>0.20.3 – tested in our GitHub action <https://github.com/DendrouLab/panpipes/tree/main/.github/workflows/integration01-ci.yml>

-	<https://github.com/scverse/scvi-tools/issues/2530> -* on jax dependencies

-	<https://github.com/scverse/scvi-tools/issues/2387> -* on model incompatibility with newest scvi-tools versions, tested in our GitHub action <https://github.com/DendrouLab/panpipes/blob/main/.github/workflows/refmap-ci.yml>

-	<https://github.com/scverse/muon/issues/135>	-* on scanpy/muon updates

- <https://github.com/scverse/muon/pull/134> -* on scanpy/muon plotting updates, fixed in our GitHub repo <https://github.com/DendrouLab/panpipes/blob/8dad1b6807cd1cffa1461e5f0d3f33a1a> 189ecd5/panpipes/funcs/[plotting.py](http://plotting.py)#L359

and tested in the GitHub action

<https://github.com/DendrouLab/panpipes/blob/main/.github/workflows/visualization-ci.yml>

To overcome such issues, we provide instructions to install the stable version of panpipes via PyPi (pip install Panpipes) and instructions to install the development versions of the pipeline by cloning the repository and installing via `pip install -e .`: <https://panpipes-pipelines.readthedocs.io/en/latest/install.html>

Critically we can now also leverage our newly introduced GitHub actions to catch these issues and fix them, as we implement new versions of Panpipes. GitHub actions is a powerful automation tool integrated into GitHub, enabling continuous integration and continuous deployment (CI/CD) directly within the repository hosting environment. We use the actions to test all the workflows implemented in panpipes with different parameters and test data.

Our implemented actions can be inspected at:

i. <https://github.com/DendrouLab/panpipes/tree/main/.github/workflows/ingestion-ci.yml>
ii. <https://github.com/DendrouLab/panpipes/tree/main/.github/workflows/filtering-ci.yml>
iii. <https://github.com/DendrouLab/panpipes/tree/main/.github/workflows/integration>-ci.yml
iv. <https://github.com/DendrouLab/panpipes/tree/main/.github/workflows/integration01>-ci.yml
v. <https://github.com/DendrouLab/panpipes/tree/main/.github/workflows/integration02>-ci.yml
vi. <https://github.com/DendrouLab/panpipes/blob/main/.github/workflows/visualization>-ci.yml
vii. <https://github.com/DendrouLab/panpipes/blob/main/.github/workflows/clustering>-ci.yml
ix. <https://github.com/DendrouLab/panpipes/blob/main/.github/workflows/refmap-ci.yml>
x. <https://github.com/DendrouLab/panpipes/tree/main/.github/workflows/spatial_ingestio>n_merfish-ci.yml
xi. <https://github.com/DendrouLab/panpipes/tree/main/.github/workflows/spatial_preproc>ess-ci.yml
xii. <https://github.com/DendrouLab/panpipes/tree/main/.github/workflows/spatial_deconv>olution-ci.yml
xiii. <https://github.com/DendrouLab/panpipes/tree/main/.github/workflows/spatial_ingestio>n_visium-ci.yml

*(7) Error messages from the pipeline are extensive and unclear. It would be beneficial to provide clearer error messages for easier troubleshooting.*

We have reviewed all our workflows and added clearer error messages to help the user with troubleshooting. The main pipeline.log outputs messages documenting the flow of tasks executed, printing out the start and completion of each job. All the tasks in each workflow send their outputs to their unique log file. To improve the overall logging throughout the pipeline, we have implemented the following changes:

1. In the main pipeline.log, we have added a new message to help the user retrieve the information about the individual task outputs. Below is an example from the integration workflow pipeline.log:

\# 2024-04-26 09:40:57,861 INFO Task enters queue = 'pipeline_integration.run_wnn'

\# 2024-04-26 09:40:57,962 INFO TASK: 'run_wnn' IN CASE OF ERROR, PLEASE REFER TO :

'logs/**4_multimodal_wnn.log**' FOR MORE INFORMATION.

\# 2024-04-26 09:40:57,963 INFO running statement: \

\#	python

/home/runner/work/panpipes/panpipes/panpipes/python_scripts/batch_correct_wnn.py	--

scaled_anndata teaseq.h5mu	--output_csv batch_correction/umap_multimodal_wnn.csv	--

figdir figures/	--n_bandwidth_neighbors 20 --n_multineighbors 200 --metric euclidean --low_memory True > logs/4_multimodal_wnn.log

2. To help users navigate the task dependencies and track their output log files, we number the logs based on the order in which the tasks are executed. In the example above, the multimodal log is prepended with the number four. Below, the output of the tree command shows the numbered log files saved after an integration workflow:

**[...]**

├── logs

├── 1_rna_bbknn.log

├── 1_rna_harmony.log

├── 1_rna_no_correct.log

├── 1_rna_scvi.log

├── 2_prot_harmony.log

├── 2_prot_no_correct.log

├── 3_atac_bbknn.log

├── 3_atac_harmony.log

├── 3_atac_no_correct.log

├── 4_multimodal_totalvi.log

├── 4_multimodal_wnn.log

├── 5_collate_mtd.log

├── 6_plot_batch_corrected_umaps.log

├── 7_lisi.log

├── batch_correction_complete.log

└── setup_dirs.sentinel

[...]

Additional examples can be found by inspecting the GitHub actions results on our repository. The actions always terminate with a tree call on the project folder for each workflow after execution to allow developers and users to verify that the expected outputs are produced.

3. We have also added a troubleshooting page to our documentation: <https://panpipes-pipelines.readthedocs.io/en/latest/usage/troubleshooting.html>

(8) *The preprocessing workflow is straightforward and effective.* We thank the Reviewer for their positive feedback on the preprocessing workflow.
(9) *The integration step is poorly documented due to the inclusion of various tools. The tutorial does not cover the full range of algorithms, leaving users to figure them out independently.*

To address this comment, we have added additional information on the supported integration algorithms, including which modalities they can be applied to, links to the references for the original manuscripts describing the algorithm development and the associated code, and links to the references for the subsequent relevant benchmarking papers. Panpipes is designed to give users a choice of algorithms that can be run and evaluated in parallel; this is critical as despite benchmarking studies the field is still using different algorithms – even for unimodal data. To give one example, inspection of single-cell studies published in the journal *Nature* in the last year reveals that at least three different algorithms (which are available in Panpipes) have been utilised, suggesting that no single algorithm may work optimally for all datasets. Therefore, we provide users with different choices, we facilitate users in finding relevant publications employing and benchmarking the different tools, and we enable decision-making by implementing metrics for integration algorithm evaluation (iLISI and the scib-metrics, which we have reimplemented in the more efficient scib-package).

Our integration documentation relating to the use of different algorithms is as follows: <https://panpipes-pipelines.readthedocs.io/en/latest/usage/integration_methods.html>

and an excerpt from this documentation is shown below:
[...]

The panpipes integration workflow implements a variety of tools to batch correct individual modalities and/or integrate across modalities to produce a reduced dimension representation of the dataset.

There are different tools available for each modality: RNA (also referred to as GEX), PROT (can be referred to as ADT) and ATAC which can be integrated into any preferred combination customising the integration workflow configuration file and running panpipes integration make full. After the results of the integration are inspected, the final object is created with panpipes integration make merge_integration.

The ideal way to run panpipes integration is to use the output MuData file from panpipes preprocess since it will already be in the required format. However, if using independent MuData the object should contain normalised data in the X slot of each modality, a ‘raw_counts’ layer in each modality, and a sample_id column in each slot of the obs and the outer obs.

Users can choose which integration method they want to apply based on their experiment, their experience with the tools or available benchmarks: we link all the relevant resources below.

[...] We offer panpipes as a framework to run multiple tools efficiently, ensuring

reproducibility of results. We believe this will empower users to choose the method that best fits their biological question, keeping a record of the hyperparameters in the configuration files, so you can safely re-run your analysis and share it with collaborators. We will continue to update the integration methods offered in panpipes and we invite you to contribute yours! The following table describes the different methods currently supported and their specificities:

| **Method** | **Type of integration** | **Modalities** | **Code** | **References** | **Benchmarks paper** |
| --- | --- | --- | --- | --- | --- |
| harmony | unimodal (batch correction) | rna, atac,  prot | harmony | Korsunsky et | Luecken et al |
|  |  |  |  | al 2019 | 2022 |
|  |  |  |  |  |  |
| BBKNN | unimodal (batch correction) | rna, atac,  prot | BBKNN | Polański et al | Luecken et al |
|  |  |  |  | 2020 | 2022 |
|  |  |  |  |  |  |
| Scanorama | unimodal (batch correction) | rna | Scanorama | Hie, Bryson, | Luecken et al |
|  |  |  |  | and Berger | 2022 |
|  |  |  |  | 2019 |  |
|  |  |  |  |  |  |

| scVI | unimodal (batch correction) | rna | scVI | Gayoso et al | Luecken et al |
| --- | --- | --- | --- | --- | --- |
|  |  |  |  | 2022 | 2022 |
|  |  |  |  |  |  |
| MultiVI | multimodal | atac, rna | MultiVI | Ashuach et al | Lee, Kaestner, |
|  |  |  |  | 2023 | and Li 2023 |
|  |  |  |  |  |  |
| totalVI | multimodal | prot, rna | totalVI | Gayoso et al | Makrodimitris et |
|  |  |  |  | 2021 | al 2024 |
|  |  |  |  |  |  |
| MOFA | multimodal | rna, atac,  prot | MOFA | Argelaguet et | Lee, Kaestner, |
|  |  |  |  | al 2020 | and Li 2023 |
|  |  |  |  |  |  |
| WNN | multimodal | rna, atac,  prot | WNN | Hao et al | Lee, Kaestner, |
|  |  |  |  | 2021 | and Li 2023 |
|  |  |  |  |  |  |

*(10) While we managed to run the unimodal batch corrections with one set of parameters, all other attempts failed.*

To ensure that different combinations of unimodal and multimodal batch corrections algorithms can be run within the integration workflow, we have now implemented continuous integration testing with GitHub actions; this allows testing different combinations of integration methods and parameters choices, by creating different configuration files. The actions demonstrate that the integration workflow is effective and we use them to ensure our code is stable with any new release to avoid the problem encountered by the Reviewer.

Integration configuration files with different uni/multimodal integrations are found below: <https://panpipes-tutorials.readthedocs.io/en/latest/uni_multi_integration/pipeline_yml.html> <https://github.com/DendrouLab/panpipes/blob/main/tests/integration_1/pipeline.yml> <https://github.com/DendrouLab/panpipes/blob/main/tests/integration_2/pipeline.yml>

GitHub actions testing the integration workflow are as follows:

<https://github.com/DendrouLab/panpipes/blob/main/.github/workflows/integration-ci.yml> <https://github.com/DendrouLab/panpipes/blob/main/.github/workflows/integration01-ci.yml> <https://github.com/DendrouLab/panpipes/blob/main/.github/workflows/integration02-ci.yml>
